# Supplementary material for: Exploring Obscurin and SPEG Kinase Biology
Source: J Clin Med. 2021 Mar 2;10(5):984. doi: 10.3390/jcm10050984 (PMC7957886; doi:10.3390/jcm10050984)
Supplement: Supplementary file 1 [file jcm-10-00984-s001.zip › jcm-1076976-supply/Supplemental File S2 - uncropped immunoblot images.pptx]

## Slide 1
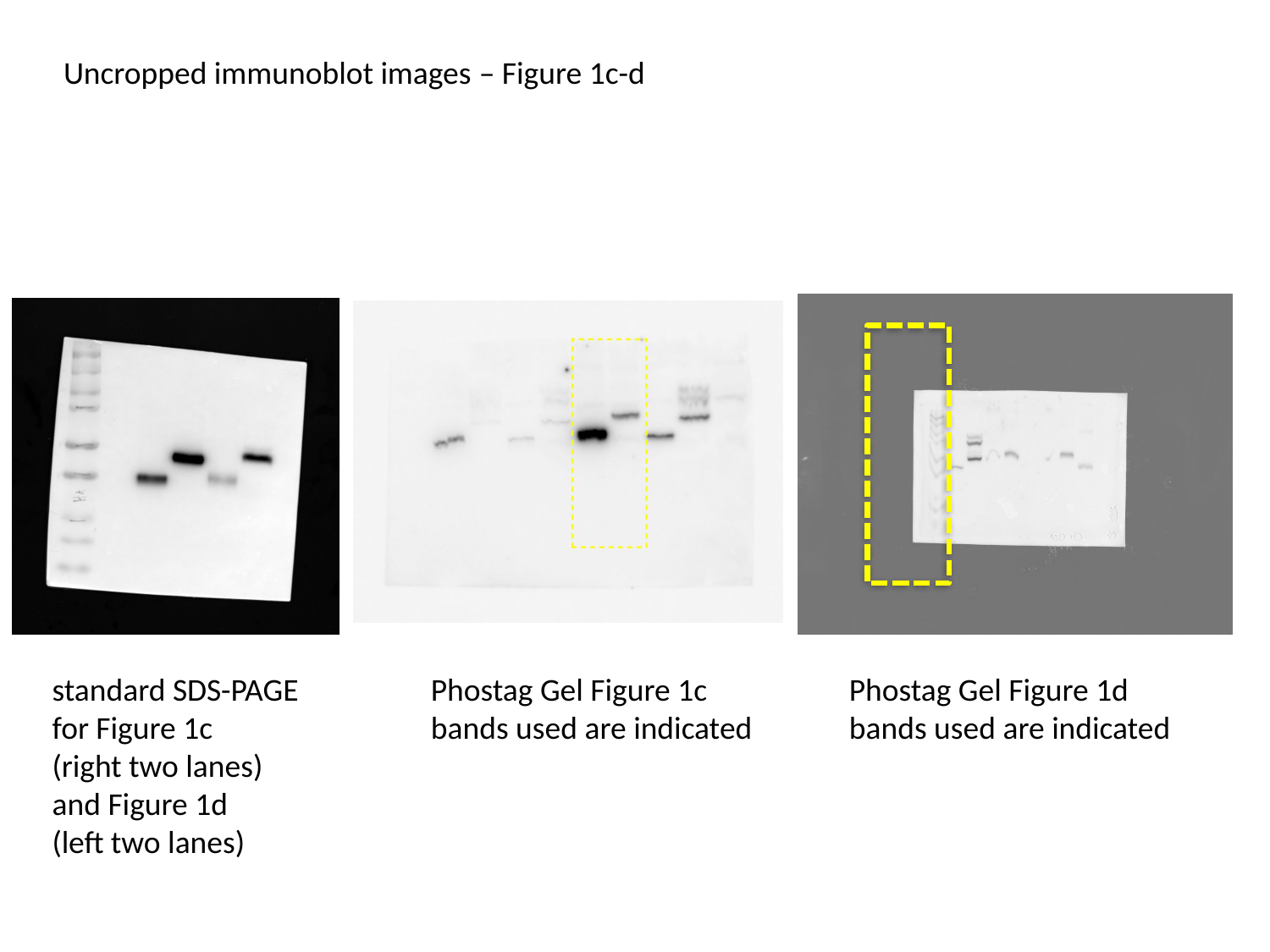

Uncropped immunoblot images – Figure 1c-d
standard SDS-PAGE
for Figure 1c
(right two lanes)
and Figure 1d
(left two lanes)
Phostag Gel Figure 1c
bands used are indicated
Phostag Gel Figure 1d
bands used are indicated

## Slide 2
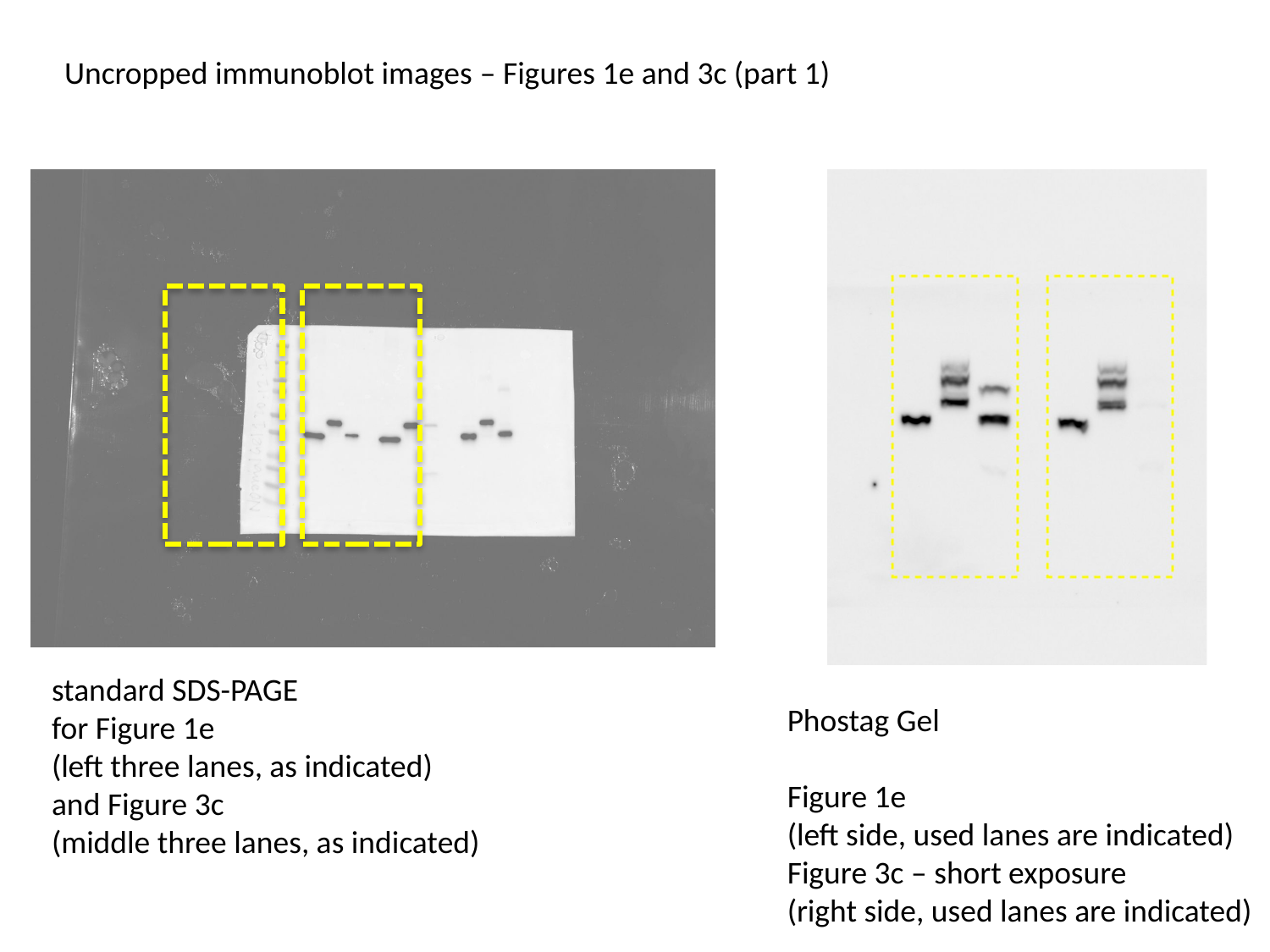

Uncropped immunoblot images – Figures 1e and 3c (part 1)
standard SDS-PAGE
for Figure 1e
(left three lanes, as indicated)
and Figure 3c
(middle three lanes, as indicated)
Phostag Gel
Figure 1e
(left side, used lanes are indicated)
Figure 3c – short exposure
(right side, used lanes are indicated)

## Slide 3
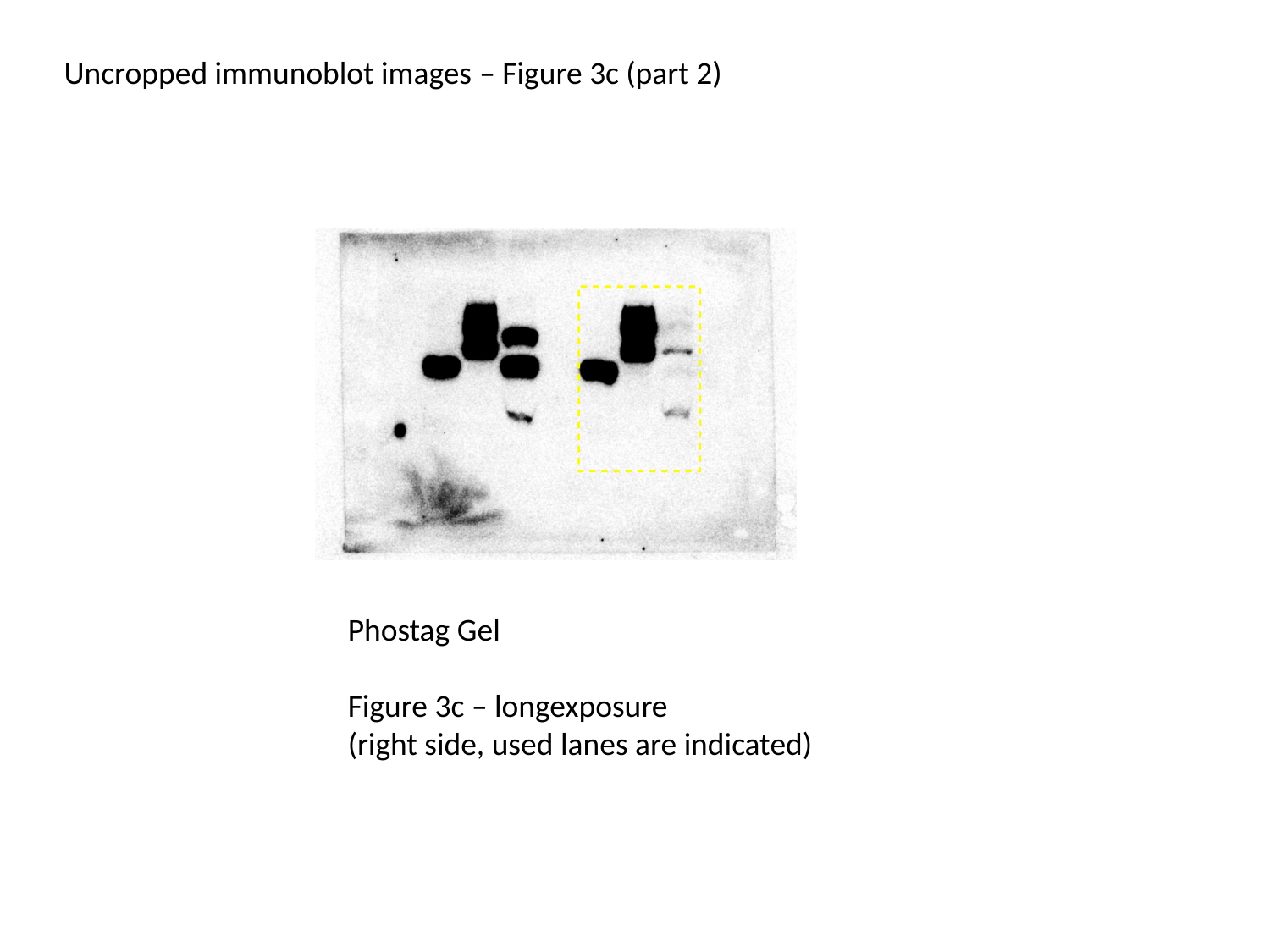

Uncropped immunoblot images – Figure 3c (part 2)
Phostag Gel
Figure 3c – longexposure
(right side, used lanes are indicated)
